# Network science for museums

**DOI:** 10.1371/journal.pone.0300957

**Published:** 2024-03-29

**Authors:** Yuji Yoshimura, Anne Krebs, Carlo Ratti

**Affiliations:** 1 Research Center for Advanced Science and Technology, The University of Tokyo, Tokyo, Japan; 2 Dominique-Vivant Denon Research Centre, Paris, France; 3 Senseable City Laboratory, Massachusetts Institute of Technology, Cambridge, MA, United States of America; University of Granada: Universidad de Granada, SPAIN

## Abstract

This paper introduces network science to museum studies. The spatial structure of the museum and the exhibit display largely determine what visitors see and in which order, thereby shaping their visit experience. Despite the importance of spatial properties in museum studies, few scientific tools have been developed to analyze and compare the results across museums. This paper introduces the six habitually used network science indices and assesses their applicability to museum studies. Network science is an empirical research field that focuses on analyzing the relationships between components in an attempt to understand how individual behaviors can be converted into collective behaviors. By taking the museum and the visitors as the network, this methodology could reveal unknown aspects of museum functions and visitor behavior, which could enhance exhibition knowledge and lead to better methods for creating museum narratives along the routes.

## Introduction

A museum is considered an informal education setting where visitors shape their experiences and construct knowledge through their movement [1–5]. The spatial layout and arrangement of the exhibits are pedagogic and aesthetic devices that create a global and local narrative; for example, as chronologically ordered exhibits are expected to be viewed in order, a strongly structured museum layout could ensure the transmission of the intended message. Conversely, special exhibits are often arranged hierarchically or architecturally for dramatic effect; for example, the *Winged Victory of Samothrace* in the Louvre is exhibited at the top of a stairway with special focused lighting. Therefore, while architectural spaces strengthen exhibition meaning, curatorial intent enhances the quality of the spatial effects and aims to improve the quality of the experience.

Although research on museum studies often stresses the importance of space and its effects on visitor experiences [6–8], Hillier and Tzortzi [9] argued that architectural intent draws less attention than curatorial intent because of the scarcity of robust tools with which to analyze the spatial structures of museums. A notable exception is *space syntax*, which provides a set of theories and techniques to quantify the spatial relationships in built environments [10,11]. Originating in architecture, methodologies were developed to quantify built environments as a system of relationships based on a *graph*. For example, a room that has more direct connections can be reached more easily and is more *integrated* into the network, but a room that requires visitors to take several steps to reach it is *segregated*. Another example is an *isovist*, which refers to the analysis of a visual field that can be seen from any single location and whose extent is measurable and is not blocked by visual obstacles such as a wall or an exhibit. This analysis enables us, for example, to determine how visitors construct their gaze when roaming in museum exhibits. Thus, the space syntax systematically measures how a space is related to all other spaces and attempts to establish the correlations using economic or social dynamics, such as pedestrian flows, land use location, crime incidents, and others [10]. In museums that use these measures and indicators, the space syntax uncovers the relationship between the museum layouts’ physical aspects and visitor behavior [9,12–15].

However, despite the success and significant contribution of this system, some aspects of the techniques and findings remain controversial in the academic community [16–20]. In addition, space syntax’s terms of expression make the interpretation of the analysis confusing, which narrows the audience. For example, Jiang & Claramunt [21] clarified that a major space syntax parameter, the *integration* index, is a normalized *closeness* centrality, which is one of the indicators being used in network analyses [22–24]. Owing to the lack of a common language, the results of space syntax’s analysis cannot be compared with those from other networks. Furthermore, other indicators developed and used in network sciences have been rarely applied, resulting in a partial rather than comprehensive analysis.

This paper aims to introduce the network science concept and methodologies into museum studies. While we acknowledge space syntax’s contributions to museum studies, we seek to explore their methodological limitations, specifically focusing on how they construct networks and apply their indicators in museum studies, which is the primary subject of our critique. We then demonstrate how our work may complement existing techniques and share fundamental values with space syntax. This study introduces a richer understanding and new tools to museum studies, the application of which enables the quantification of the spatial graph to reveal its properties, which can better help curators and museum professionals organize exhibitions. Additionally, our work can benefit network science communities because researching networks in *space* is considered an emerging field in the physics of complex networks [25]. As spatial networks are embedded in two- or three-dimensional Euclidean space, they belong to a network type that is different from non-spatial systems, which are the focus of network science. Therefore, researching networks for museum studies requires a different approach to network science, as the nature of a museum building is *temporal-spatial*. Moreover, applying network science to spatial networks in museums elucidates the unknown aspects, constraints, and limitations of spatial properties, resulting in augmented knowledge in network sciences.

## Theoretical background

Network science has been called “the science of the twenty-first century” [23, p.25] and “the science of the real world–the world of people, friendships, rumors, disease, fads, firms, and financial crises” [26, p.13]. It has been widely used in many disciplines such as physics, biology, computer sciences, engineering, and economics (for example, see Estrada, Fox, Higham, & Oppo [27] for its application in a range of disciplines). Network science analyzes the relationships between components rather than analyzing the features associated with each individual component to understand how individual behaviors are converted into collective behaviors in a bottom–up way as self-organized systems. Consequently, striking regularities emerge among seemingly different phenomena, observing similar patterns and scaling laws.

Network science arose from graph theory and sociology [23], with the former being used to mathematically research graph properties. Dating back to the 18^th^ century to Euler’s seminal work in the 1730s on the Konigsberg bridge [28], graphs provide a better and more intuitive visual method for dealing with abstract concepts such as the relationships of individuals within a network. As graph theory is able to quantify aspects such as similarity, hierarchy and network efficiency in many other disciplines, it has become an extensive and popular branch of mathematics. The association between network science and sociology is because sociology focuses on the non-spatial nature of social links by analyzing the relationship between the actors in a system (see Wasserman & Faust [29] for an overview) in an attempt to quantify through distance measures how people’s relationships affect their behaviors. Freeman [30] described some early sociological research on social networks.

Museum research, however, has explored networks based on a museum’s physical structure or on visitor behavior. For example, Kanda et al. [31] assessed sequential visitor movements between locations using radio frequency identification that was attached to the exhibits and used by their visitors. Although they could successfully extract patterns, the spatial museum structure was relatively simple, and the visitor trajectory was in a one-way clockwise direction. Yoshimura, Krebs, & Ratti [32], Yoshimura, Sinatra, Krebs, & Ratti [33], and Yoshimura et al. [34] considered the structure of a large-scale museum as a network and collated large-scale datasets of sequential visitor movements between locations at which Bluetooth sensors were installed, from which the most-used trajectories and visitor path similarities were deduced from the length of the museum visit.

However, the most widely used concepts and techniques for museum network research have been related to space syntax [7,9,15]. It is based on the following concept: “the way spatial layouts are used and how they function is not only about the properties of individual spaces, but about the complex relations between spaces and how they affect each other by co-existing simultaneously–defined as configuration in syntax (Hillier and Hanson, 1984; Hillier, 1996)” (Tzortzi [7], p.103-104). For example, to describe the properties of each space, they use the concepts of *connectivity*, which counts the number of direct links from a specific node, and *space types*, which describe how each space is embedded in the layout [10].

**Fig 1:**
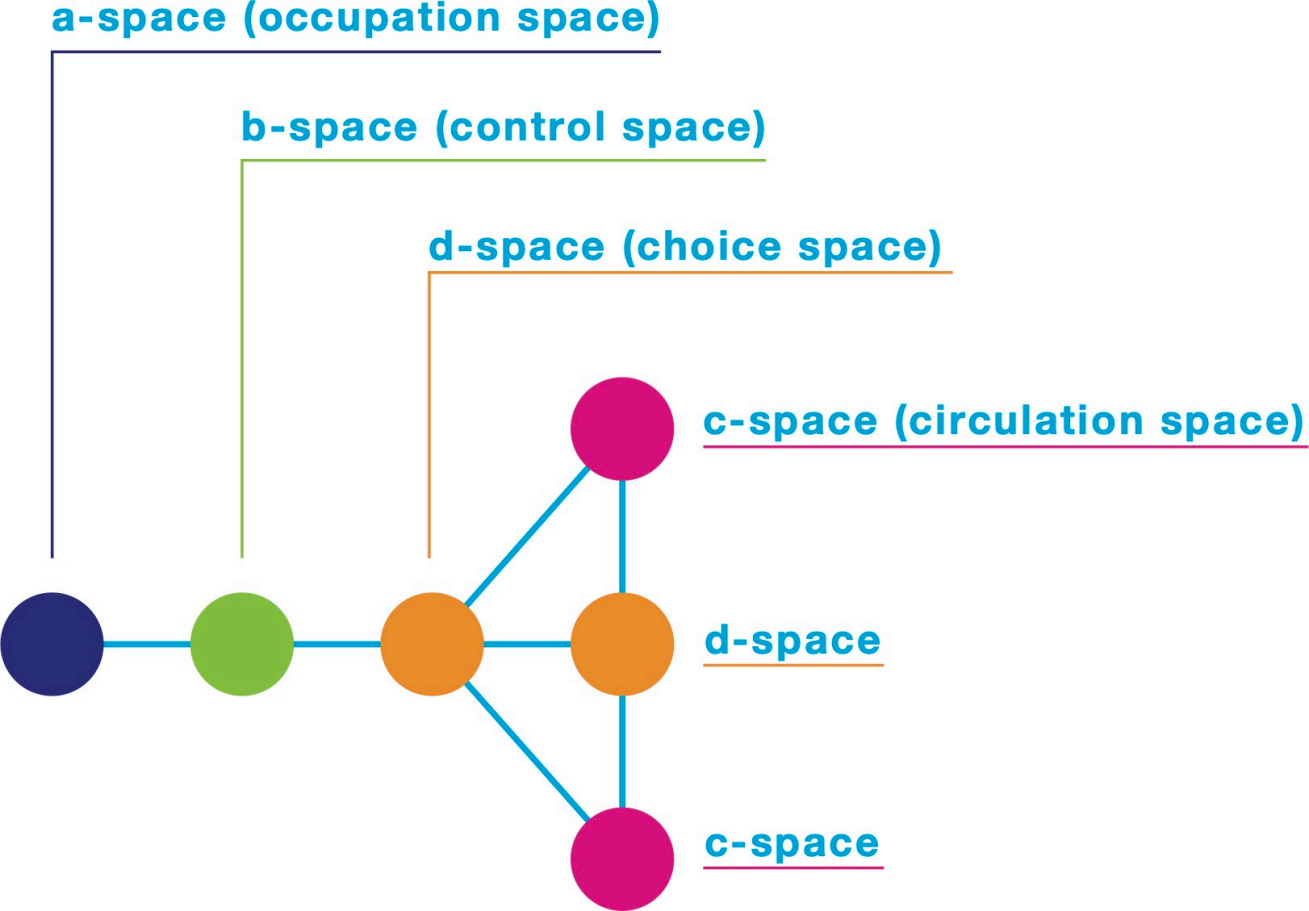
Space types proposed by Hillier [10]. The author of this paper made this illustration based on Fig 3 in Tzortzi [35].

Table 1 and Fig 1 illustrate Hillier’s [10] proposed space-type concept, which is widely used with other concepts such as *depth* and i*ntegration*. A room’s *depth* score is determined by the number of steps people need to take from a given origin (the room) to all other points; that is, each room in the system is numbered based on the number of spaces they are from a specific origin. All scores are then summed to determine the *total depth* of an origin from all other rooms, which is the basis for measuring the *integration* value of each space in the building; that is, the smaller the *total depth*, the greater the room integration in the building, the fewer rooms a person needs to pass through to visit the entire building, and the greater the room integration in the whole network.

**Table 1:** Space-type definitions (from Table 1 in Tzortzi [35]).

	Space Type	Definition
a	occupation space	a-spaces are dead-ends, so cannot be passed through
b	control space	b-spaces control access to a-spaces (or other b-spaces) and so offer only the same way back
c	circulation space	c-spaces form rings, so offer one alternative way back
d.	choice space	d-spaces offer more than one alternative way back, so route choices

However, as described in the introduction, space syntax methodologies have been found to apply only to the space syntax discourse. For example, *connectivity*—which we introduced as a space syntax concept—is known as *degree* in network science, “to-movement” or *integration* is known as closeness centrality, and “through-movement” or *choice* is known as betweenness centrality. Therefore, “these properties of ‘integration’ and ‘choice’ are essentially synonyms of ‘closeness centrality’ and ‘betweenness centrality’ in network theory, and they are confusingly renamed within the field of space syntax” [36]. Because of syntactic differences, it is difficult to compare museums’ network properties and visitor behavior with the results of other museum network studies.

## Methodology

There are several ways to convert a spatial structure into a graph. In this paper, the primal representation [37] was applied, where the centroid of the room is the node, and the center line of the corridor or any kind of walkable connection between the rooms is the link. The undirected graph *G* has two elements, *N* nodes and *E* edges (links), between each pair of nodes. Graph *G* is described using an N*N adjacency matrix **A**, whose entry *a_ij_*(*i*, *j* = 1, …, *N*) equals 1 if there is a link between node *i* and node *j* and 0 otherwise. The network can also be weighted; while in the unweighted network, each link has an equal weight (*a_ij_* = 1), in a weighted network, each link has a different weight such that the link (*i*,*j*) has the weight *w_ij_; a_ij_ = w_ij_*. For example, in a museum, the network can be weighted in terms of room size, corridor length, or the number of doors/entrances.

The concepts associated with path and path length play important roles in network science, where the path is the route that follows the links, and the path length is the number of links on the route. The following describes some of the key concepts and ideas behind network science and considers an appropriate framework for museum studies.

**Fig 2:**
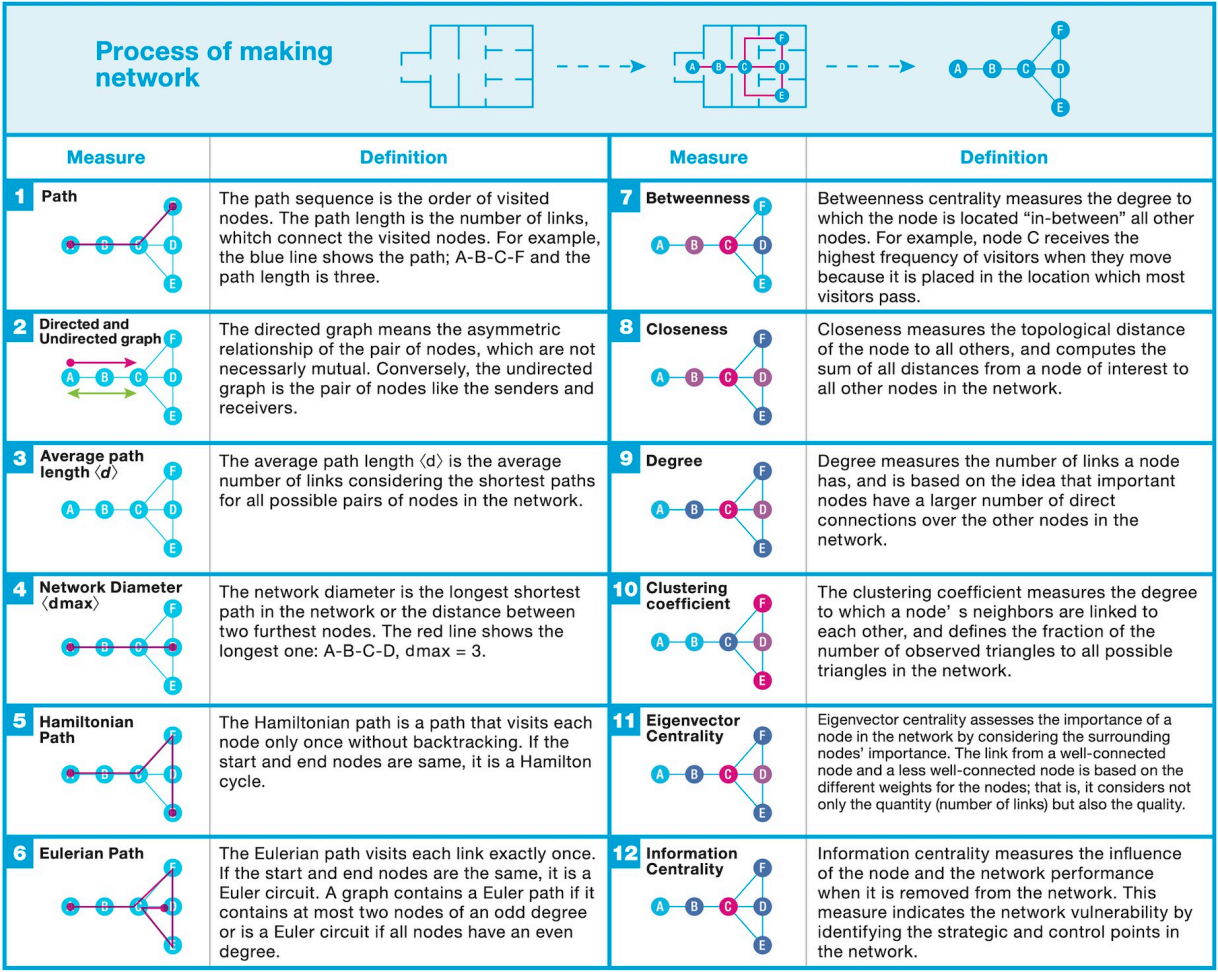
Key concepts for graph theory and network science. The figures in the left columns were developed by the author of this paper, based on Fig 2.13 in Barabási [23].

Fig 2 shows some diagrams for some of the key network properties. The first row of Fig 2 describes the process for converting a museum plan into a primal representation of that museum (= *G*), for which there are 6 nodes (*N* = 6) and 7 links (*E* = 7). Although these are the basic and fundamental properties of networks, it is possible to apply them to a museum situation and interpret the results in the context of museum studies. For example, a visitor’s sequential movements can be described as a path composed of the order of visited nodes and the links between them. The shortest path between node *i* and node *j* (*d_ij_*) is the route that has the least links in the network, and there are several shortest paths for a pair of nodes that do not have a loop. In the undirected graph this is represented by *d_ij_ = d_ji_*. The network diameter (*d_max_*) is the maximum length of the shortest path for all node combinations in the network. The average path length 〈*d*〉 is the average number of steps for all possible pairs of network nodes, which gives a general idea of how many links a visitor needs to visit the entire network.

One of the key characteristics of the node can be described using the *degree* indicator, which is based on the idea that an important room would have a larger number of direct connections than the other rooms in the network. Therefore, based on the number of links to a node, the degree reveals the room hierarchy. *Degree distribution* (*p_k_*) is frequently used to analyze the collective behaviors of the network, with the function type *p_k_* largely determining the network properties. For example, a few rooms could have many links, which would make them network hubs, while the majority of rooms may have only one link. However, although the *degree distribution* provides significant information (e.g., the existence of the hub), it does not explain all network aspects, and gives little information on the surrounding and neighboring nodes. The *clustering coefficient* (*C_i_*) measures the degree to which a node’s neighbors are linked to each other; that is, if node *i* has no link, *C_i_* = 0, and if node *i* has links with all other neighboring nodes, *C_i_* = 1. A “small world” [38] can be discovered when the average topological distance between nodes is small compared with the size of the whole network, which indicates a local circulation on a neighborhood rather than a global scale in the whole network.

The *degree correlation* also provides information about a node’s links to other nodes. For example, nodes that have similar degrees tend to have a similar number of links to each other, resulting in the positive *degree correlation*, which is known as an “assortative network.” Conversely, a node that has a larger degree and links to smaller nodes results in negative *degree correlation*, which is known as a “disassortative network.”

Apart from the properties already mentioned, several other graph theory properties could be useful for museum studies. The *Hamilton* cycle indicates a graph in which all nodes are visited exactly once and the start node coincides with the end node, and the *Eulerian* graph is where all links are visited exactly once but permits the same nodes to be revisited; if the start and end nodes are the same, it is known as a *Euler* circuit. These graphs imply that a museum with these properties would be efficient in terms of transmitting its intended message to visitors; that is, because there would only be one determined sequence, visitors would have no option but to take this as the structural properties would not allow visitors to randomly explore the museum.

### Centrality indices

Social network analysis (SNA) has several indicators that allow for the quantification and analysis of the centrality in network science [22,39]. In this section, in addition to the *Degree* and *Clustering Coefficient*, four important centrality indicators that measure the centrality of spatial networks are described: *Betweenness* centrality (*C^B^*), *Closeness* centrality (*C^C^*), *Eigenvector* centrality (*C^E^*), and *Information* centrality (*C^I^*). Complete definitions for these indices can be found in Porta et al. ([37], pp.706-10) and a detailed description of SNA can be found in Valente [40].

*Betweenness* centrality measures how frequently node *i* acts as a mediator on the shortest paths between a pair of nodes in the network. A node with a higher degree of *betweenness* centrality has a higher performance; that is, it passes through a node or traverses the shortest paths, with the intermediate node acting as a strategic control point. *Closeness* centrality measures the inverse of the average distance from node *i* to all other nodes and is determined by the number of edges when traversing from node *i* to node *j*. *Closeness* centrality has been widely used in urban, regional, and transportation planning to assess access.

*Information* centrality (current flow closeness) measures the importance of node *i* to optimum network functioning; that is, it identifies those nodes that, if removed, would be the most disruptive to network performance; therefore, node *information* centrality can be quantified by the effect on network performance following deactivation (e.g., dropping node *i* from the network). This indicator can also be considered a variance of the *closeness* centrality; however, the difference is that it counts the number of shortest or relatively short paths between the nodes that could work as alternative routes to spread information in the network if a certain node was removed. *Eigenvector* centrality is a weighted version of *degree* centrality and assesses node *i*’s significance in the network based on the importance of its neighbors; that is, the links from the important nodes are considered more significant than the links from the unimportant nodes. Therefore, this indicator considers not only the number of connections a node has, but also the weight of the vertices it is connected to. In this way, it can measure the influence of a node based on the surrounding influential nodes in the network.

## Results

For this study, five spatial networks were analyzed: the two theoretical networks from Hillier and Tzortzi [9], which we demonstrate can be further classified using centrality indicators, and three large-scale museums: the Louvre Museum, the Metropolitan Museum of Art (the Met), and the Museum of Fine Arts, Boston (MFA). The visitor floor plans act as the data sources for each of the three respective museums. After this, and using the previous section’s methodology as a basis, we manually created our network for analysis.

### Case study 1: Theoretically generated extreme layout

The basic properties of each network are described in Table 2. Under a single-ring layout, visitors must take slightly more steps to visit the farthest rooms in the network than under a grid layout. The average number of necessary steps for the shortest path is larger for the single-ring layout than for the grid layout (3.76 vs. 2.59).

**Table 2:** Basic properties of spatial layout.

	Nodes	Edges	*d*	〈*k*〉	〈*C*〉	Network Diameter
Single ring of spaces						
	14	14	3.76	2	0.00	7.00
Fully connected grid						
	17	28	2.59	3.29	0.10	5.00

However, the average degree 〈*k*〉 is larger for the grid layout than for the single-ring layout (3.29 vs. 2.00). As the single-ring layout has no local connectivity (〈*C*〉 = 0), there are poorer connections between the rooms. This suggests that there are fewer spatial choices for visitor movements.

**Fig 3:**
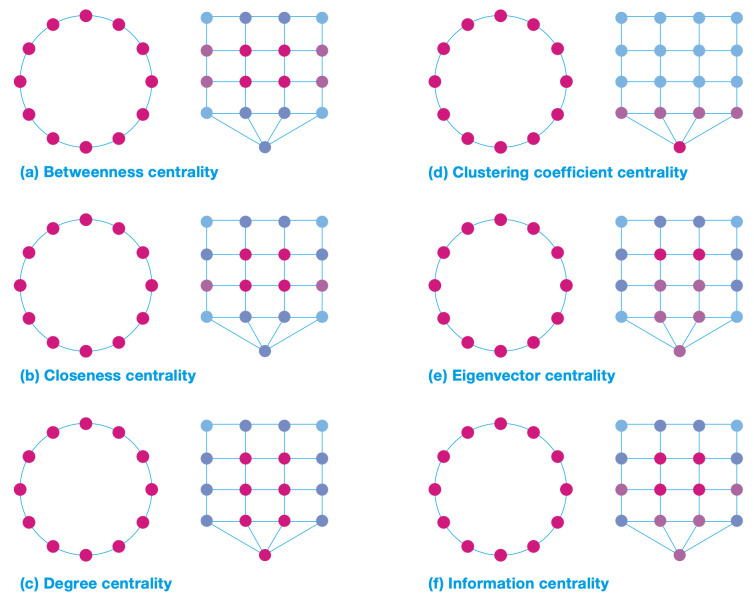
Computation results for the centrality indicators. (a) betweenness, (b) closeness, (c) degree, (d) clustering coefficient, (e) Eigenvector, (f) Information.

Fig 3 shows the results of the computation for the centrality indicators for both datasets. The centrality scores for all indicators do not change for the single large ring layout; that is, the node score is independent of the type of indicator.

The main reason for this result is related to the graph structure; because each node has two links, identical results are generated for every node. Therefore, a strongly sequenced structure could make the same people stay together from the entrance to the exit, which would decrease the probability of their having informal encounters with others.

However, the results for the grid layout change significantly depending on the centrality indicators. Higher *betweenness* scores can be found in the four nodes at the heart of the network, which means that although visitors are free to explore any rooms in the network, they are required to pass through these rooms to take the shortest paths. Similarly, higher *closeness* scores are found in the heart of the network and decrease gradually toward the periphery, which indicates that the former rooms are close to all the other rooms in the network and, consequently, they are more “integrated” than the ones on the periphery. The *information* centrality also indicates that those nodes are key for overall network performance, suggesting that if these nodes were removed, the network would have a communication problem.

The higher scores for *degree* create a central circulation space network of routes from the entrance, which indicates that those rooms have a larger number of links to other rooms and a higher potentiality for movement choices, but does not show the importance of the links based on where they come from. The *eigenvector* centrality, however, indicates the hierarchy of the nodes, as this metric assesses the importance of the nodes that a link is coming from and is therefore able to differentiate the importance of nodes in the network. The *clustering coefficient* measures the potentiality of the local circulation to count the triangles connecting each room. For example, the route through the entrance creates a change of direction and the possibility to backtrack and loop back, which includes the main circulation or the internal axis in the local cluster. The hierarchical spatial organization identifies the visit circulation between the rooms.

Therefore, as demonstrated, the centrality indicators are able to further classify the original spatial types [9] and reveal the hierarchy. For example, *information* centrality measures the whole network performance when a node is removed, which, while being an extension of *closeness* centrality and the *integration* index, can shed light on different aspects of the network. Therefore, the centrality indicators provide information about the node on both local and global scales.

### Case study 2: Application to large-scale museums

Table 3 presents the basic properties of the large-scale museum networks (i.e., Louvre Museum, Metropolitan Museum of Art, and Museum of Fine Arts, Boston).

**Table 3:** Basic properties of three large-scale museum networks.

	Nodes	Edges	*d*	〈*k*〉	〈*C*〉	Network Diameter
Louvre Museum						
	432	535	14.7	2.477	0.064	39.0
Metropolitan Museum of Art						
	462	656	9.254	2.84	0.116	21.0
Museum of Fine Arts, Boston						
	238	297	8.069	2.496	0.028	19.0

The Louvre and the Met have a similar number of nodes, while the MFA has almost half as many. The Met has 22% more links than the Louvre, but only 0.7% more nodes, which suggests that the Met would have larger *degree* scores than the Louvre. Moreover, we discovered that many of the rooms inside the Louvre have less than three links. The MFA has almost the same average degree 〈*k*〉 scores.

However, there are significant differences in network diameter, with the Louvre having a much larger network diameter (39.0) than The Met (21.0), which is only slightly higher than the MFA (19.0). This is counterintuitive because there is a significant difference between the number of nodes and links at The Met and the MFA. On checking the similarity in *d*, both museums were found to require a similar number of steps on the shortest path, while the Louvre needed around 14 steps; that is, while the MFA has almost half the number of rooms as The Met, there is only a one-step difference in the average length of the shortest path.

**Fig 4:**
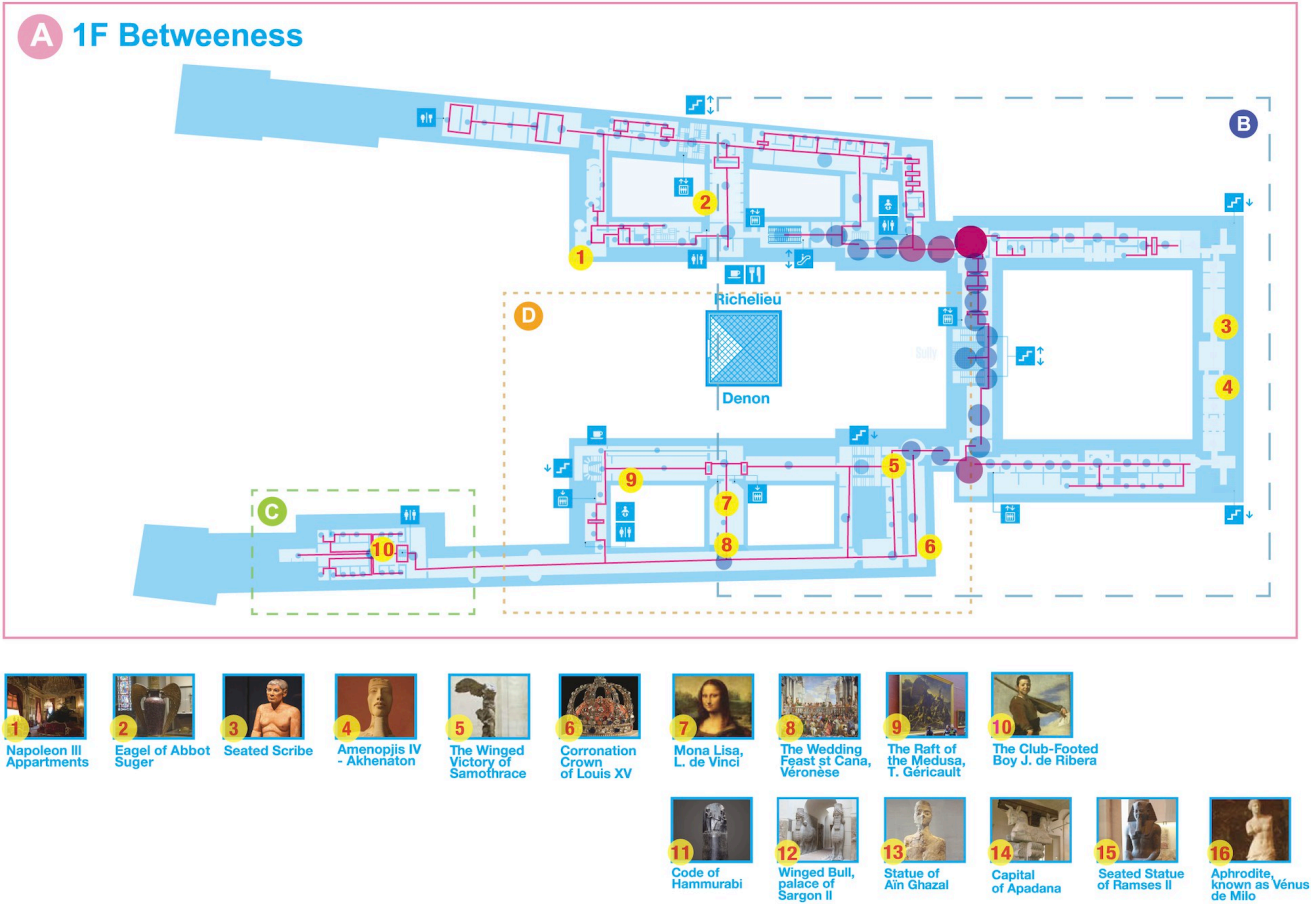
Topological representation of the spatial structure of the first floor of the Louvre Museum. The nodes identified by numbers indicate the most popular or busiest places or works of art. The size of the node indicates the centrality (the higher the score, the larger the node).

Fig 4 presents an example of the application of centrality indicators to the Louvre Museum, in which the letters indicate the popular artworks/places, and the size of the dots indicates the centrality measures.

The glass pyramid has one of the highest *betweenness* scores, indicating that this place receives a higher frequency of visitors as they move between the origin and destination pairs in the museum. That is, the glass pyramid acts as a central location. These results add one more aspect: the current congested situation at the glass pyramid may derive not only from its function but also from its structure. For example, hypothetically, if the glass pyramid did not have an entrance function, visitors would still gather there because they are required to pass by it due to the spatial structure. Additionally, the *Mona Lisa* room is located on the periphery in terms of *betweenness* centrality, suggesting that the current hyper-congested situation may be largely created by the attraction power of the exhibit rather than the spatial structure (see Fig 4, A, 1F Betweenness).

**Fig 5:**
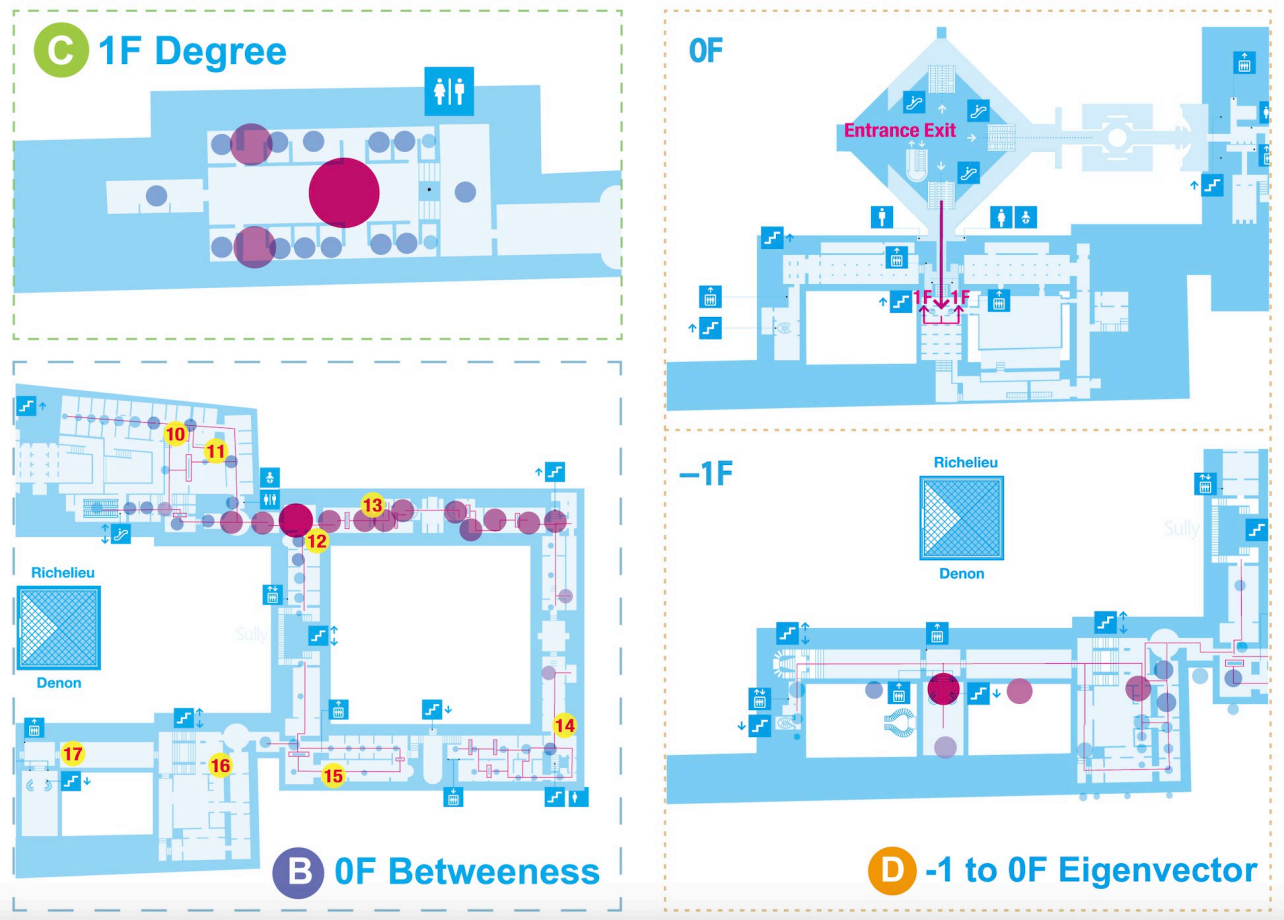
Topological representation of the spatial structure of the basement, ground floor, and first floor of the Louvre Museum. The nodes identified by numbers indicate the most popular or busiest places or works of art. The size of the node indicates the centrality (the higher the score, the larger the node).

Conversely, the “Porte des Lions” secondary entrance shows the highest score for both *degree* and *clustering coefficient* centrality (see Fig 5, C, 1F Degree). As this entrance is located at the end of the Great Gallery, which is the farthest from the glass pyramid, it seems that it would be isolated and form an independent sequence, an assumption that is further strengthened by the lower other global centrality indicators (betweenness and closeness). The room behind the great staircase toward The Winged Victory of Samothrace has the highest *degree* centrality, possibly because this room is a key connection between the Denon and Sully wings toward the Venus de Milo, which also has a relatively high degree centrality.

The *degree* and *clustering coefficient* centralities enable the local network properties to be measured. However, as they identify the same weights when counting a link from the glass pyramid and one from a tiny room, *eigenvector* centrality is used to assess the value of the links, and it indicates a sequential path from the Denon entrance to the Daru gallery toward the *Venus de Milo* (see Fig 5, D, -1 to 0F *Eigenvector*), which largely coincides with the visitors’ most-used path [34]. Therefore, the visitors’ actual movement and their paths allow for a visualization of the room hierarchy in terms of the others that they are linked to.

However, the most interesting and relevant results for museum management may be the *information* centrality because it shows the degree of network performance when a node is eliminated from the network, and can therefore highlight those nodes and links that are fundamental to network function. While these components may not be the most connected or have the largest number of links, they need to be monitored and protected in urgent situations. Although the top two highest scores are not surprising (the entrance room at the Sully Wing from the glass pyramid, and the glass pyramid), two other critical components of the Louvre’s infrastructure network were identified: the escalator space in the Richelieu Wing and the stairway space in the Sully Wing. Although the main function of these areas is to provide vertical connections between the floors, these results indicate that they are also fundamental to visitor circulation throughout the building.

## Discussion

This paper demonstrated that network science methodologies are able to successfully capture spatial museum properties more efficiently than previously suggested methods [9–11] and are able to reveal a greater range of museum spatial characteristics. Further, as the methodologies demonstrated in this paper are based on standard network science measures [23], it is possible to compare the obtained results with results from other museum networks by other researchers from different disciplines. In this section, we discuss the implications of the results in the framework of museum studies.

Rooms with higher *betweenness* scores attract a higher number of passing visitors because these visitors are required to pass through those rooms when they move between the origin and destination pairs. As the attracting power of *betweenness* is independent of the exhibit or the way it is displayed, this is significant for museum professionals because this spatial feature can be employed to create more effective, strategic curatorial intent. For example, a popular exhibit could be located away from a “central” or “over-crowded location” to decrease museum congestion, as can be seen at the Louvre, where the *Mona Lisa* is located on the “periphery” in terms of *betweenness* rather than in a central place to reduce congestion and multiply the gathering effect already caused by the attraction power of the exhibit [32,34]. Conversely, a higher *degree* indicator indicates a larger number of direct connections to other rooms, which increases visitor spatial choices. Rooms with more direct connections are at the top of the hierarchy in the building; therefore, the distribution of *degree* suggests the room hierarchy. *Eigenvector* centrality can be considered a variation of *degree* centrality; however, it places more value on links coming from important nodes. Consequently, this indicator also reveals the room hierarchy by considering the influences of the surrounding and important rooms. Therefore, this type of metric could be useful for museum studies when seeking to construct a hierarchy of intended messages and routes. For example, a highlighted exhibit could be strategically placed in a room that is higher in the hierarchy, making it appear more impressive. However, *degree* and *eigenvector* cannot specify a location if it is at the heart of the network or on the edge, as depending on its location, the role of each room is significantly different; for the former, the room would play an important role in facilitating a global circulation that would distribute visitors to the peripheral connections, while the latter works to facilitate a local circulation on the periphery.

Higher *clustering coefficient* indicator scores indicate rooms that are locally interconnected, which could be used to enhance and encourage local circulation in a specific neighborhood. Therefore, these rooms could have a controlling function for organizing local circulation, act as transition spaces from the main axis to the local cluster, and play a secondary role in the display function in terms of pedagogical function. For example, separate themes from the principal narrative could be organized among local neighbors, creating a local focal point. In addition, resting areas between exhibition rooms could be created to organize isolated episodes, encourage reflection, and stimulate social interactions. Therefore, this property introduces spatial choices into local visitor itineraries without losing the sense of the whole sequence.

A higher *closeness* indicator score indicates that the room can be reached from all others in fewer steps, implying that visitors would gather more easily in an *integrated* space than in a *segregated* space, and an exhibit placed in the latter space would have a lower probability of being viewed by visitors. Therefore, this indicator could be used, for example, to create *segregated* spaces devoted to more specialized collections or more specific themes and could attract specific visitors to less-visited rooms by offering a different spatial and aesthetic experience; further, a popular exhibit could be placed in a *segregated* room rather than in a central location in the museum, which could more evenly distribute the visitors across the spaces and reduce potential congestion. *Information* centrality is a variation of the *closeness* indicator that measures a network’s performance if a node is removed from the network, which could be very helpful for museum professionals when planning partial closures in preparation for a temporary exhibition or renovations. If the museum needs to close a room with a higher score, museum professionals could improve alternative routes and communications between the other rooms.

All of these facts validate the applicability of network science to museum studies. The proposed methodology, derived from network science, provides effective tools to measure spatial properties, with their implications being useful for curators and museum professionals either for security or for interpretation strategies. For example, if curatorial intent attempts to transmit a unique message, the *Hamilton* path or *Eulerian* path would be helpful as an exhibition structure because those paths can ensure unique sequential movements to direct visitors toward a path in the same order as the curatorial intent. In contrast, if the curatorial strategy is to encourage visitors to explore the space and facilitate encounters between visitors and exhibits, the curator would consult the *betweenness* indicator to develop the proper strategy, which would increase the probability of encounters and re-encounters. The *closeness* centrality could also be used to place a key highlighted artwork in a recurrent gathering room in a hierarchal way or make less popular exhibits more visible to visitors via their spatial properties.

Thus, the proposed method provides clear value and novel perspectives to the existing research, but it also has some limitations.

Our analysis and proposed methodology cannot reveal any qualitative information, such as the visitor’s decision-making process or value consciousness, including their psychological factors. To uncover these aspects, we must assess the traditionally conducted data collection methodology, such as interviews, questionnaires, or participatory observation [41]. Additionally, this paper does not consider the influence of museum guides, orientation signs, and audio guides on visitors’ decisions to explore the museum. We consider that all of them would probably affect visitors’ paths and their duration of stay at the specific locations [32,34]. Thus, we consider them for our future works.

Overall, network science provides strong tools for museum professionals to uncover the different aspects of the museum and visitor behavior and can enhance the application and knowledge of network science. Because buildings are a different system class because of their *spatial* features, the application of network science to museum studies requires a different approach than non-spatial systems [25]. This means that some previous findings in network sciences (e.g., small-worlds, scale-free properties) cannot be fully compared with a museum’s network properties. This is mainly because the spatial network is embedded in the real space, where nodes are embedded in a two- or three-dimensional Euclidean space, and edges do not define relations in the abstract space. Consequently, spatial embedding strongly restricts topological analysis, significantly affecting the observation of small-world or scale-free degree distributions, which are discovered as properties of other networks. Conversely, this means that any museum studies based on network science would add new knowledge in both the network science and museum studies fields.

This paper demonstrated that museums represent interesting, innovative, and complex networks and revealed the possibilities for mutual collaborations between network science and museum studies. More precisely, the proposed methodology, which views museum studies through the network science lens, enables us to better compare and research the spatial properties of museums and visitor behavior, which could be extremely useful to curators seeking to enhance global and local museum narratives and increase the quality of museum visitor experiences.

## Supporting information

S1 Data Louvre museum network data. (CSV)

S2 Data Metropolitan museum of art network data. (CSV)

S3 Data Museum of fine arts Boston network data. (CSV)
